# Cytokinin but not gibberellin application had major impact on the phenylpropanoid pathway in grape

**DOI:** 10.1038/s41438-021-00488-0

**Published:** 2021-03-01

**Authors:** Kamal Tyagi, Itay Maoz, Bettina Kochanek, Noa Sela, Larry Lerno, Susan E. Ebeler, Amnon Lichter

**Affiliations:** 1 Department of Postharvest Science, Agricultural Research Organization, The Volcani Center, Rishon LeZion, Israel; 2 Department of Viticulture and Enology, University of California, Davis, CA 95616 USA; 3 Department of Plant Pathology, Agricultural Research Organization, The Volcani Center, Rishon LeZion, Israel; 4 Food Safety and Measurement Facility, University of California, Davis, CA 95616 USA

**Keywords:** Cytokinin, Gibberellins, Secondary metabolism

## Abstract

Cytokinin and gibberellic acid (GA) are growth regulators used to increase berry size in seedless grapes and it is of interest to understand their effects on the phenylpropanoid pathway and on ripening processes. GA_3_ and synthetic cytokinin forchlorfenuron (N-(2-chloro-4-pyridyl)-N′-phenylurea, CPPU) and their combination were applied to 6 mm diameter fruitlets of ‘Sable Seedless’, and berries were sampled 51 and 70 days (d) following application. All treatments increased berry size and delayed sugar accumulation and acid degradation with a stronger effect of CPPU. CPPU, but not GA, reduced berry color and the levels of anthocyanins. While CPPU reduced the levels of anthocyanins by more than 50%, the combined treatment of GA+CPPU reduced the levels by about 25% at 51 d. CPPU treatment had minor effects on flavonols content but increased the levels of monomeric flavan-3-ols by more than two-fold. Phloroglucinol analysis using HPLC showed that proanthocyanidin content was significantly increased by CPPU, whereas mean degree of polymerization was reduced from 26 to 19. Volatile analysis by GC-MS showed changes in composition with CPPU or GA treatment with potential impact on flavor. RNA-seq analysis showed that GA had a minor overall effect on the transcriptome whereas CPPU had pronounced effects on gene expression at both 51 and 70 d. Comparing the control and CPPU at similar Brix of ca. 19.7°, a reduced expression of *stilbene synthases* (STSs) including their regulators *MYB14* and *MYB15*, and other phenylpropanoid-related genes was observed in CPPU-treated grapes. Overall, our study shows that CPPU had a major influence on the phenylpropanoid pathway and affected multiple ripening-related processes.

## Introduction

Fruit development is mediated by plant growth regulators (PGRs) that control its major developmental processes^1^. As grape berries develop, they exhibit a double sigmoid growth curve separated by veraison that marks the beginning of ripening^2^. Cell division and expansion are the major events during the first phase and are accompanied by synthesis and accumulation of organic acids, methoxypyrazines, and phenolic compounds such as proanthocyanidins (PAs) and hydroxycinnamates. This phase of berry growth is under the control of auxins, gibberellins, and cytokinins. Auxins and gibberellins are mostly produced by the seeds while the source of cytokinins in fruits is less established and it is likely to be imported from the plant^2^. The number of seeds in the berry can determine berry size, and the lack of seeds or the presence of unfertilized ovules in sternospermocarpic (‘seedless’) berries can be partly complemented by external gibberellin^3^. Schematic representation of the levels of PGRs during development suggests that auxin levels are high at berry set and decrease during phase 1 while cytokinins and gibberellins peak during phase 1^2^. The second phase, verasion, marks the beginning of major processes in grapes ripening, berry softening and anthocyanins accumulation in colored varieties. There are reports of a small peak of ethylene preceded by a large peak of abscisic acid (ABA) which coincide with veraison^4^. Brassinosteroids increase at veraison and participate in ripening, possibly by modulation of ethylene content^4^. On the other hand, auxin treatments retard sugar and anthocyanin accumulation and prevent the decrease in acidity and chlorophyll concentration, and also cause a delay in the usual ripening-associated increase in the levels of ABA^5^. The third phase in berry development is ripening which is characterized by accumulation of glucose and fructose, as well as a decrease in the levels of organic acids. Volatile compounds that are produced by grape berries during development and ripening include fatty acid derivatives that are the most abundant group, monoterpenes that are prominent flavor compounds in Muscat flavored grapes, sesquiterpenes, C_13_ norisoprenoids, volatile sulfur compounds, and methoxypyrazines^4,6,7^. Some volatile compound types such as methoxypyrazines and sesquiterpenes, C_13_ norisoprenoids and volatile fatty acid derivatives accumulate in the berry before veraison while volatile monoterpenes and volatile sulfur compounds accumulate during ripening^4,5^.

Physiological studies on the role of PGRs in fruit development often rely on external applications of the hormones or their agonist followed by observations of the changes in fruit development. In table grapes there is a plethora of applicative studies aimed at increasing the berry size in ‘seedless’ varieties and the color of red varieties grown in hot regions^8^. The effect of GA was studied from the late 1950s^9^ with timing and concentration being major factors. In ‘seedless’ grapes, application of GA to increase berry size is performed at a fruitlet diameter of 4–6 mm because earlier application can have negative impacts on fruit-set and berry shot (berry set too high and presence of very small berries) and later application is less effective. The effective concentration of GA can vary markedly according to sensitivity of the variety to the hormone. For example, ‘Thompson Seedless’ requires multiple applications and large cumulative amounts of GA exceeding 100 ppm, while a single application of 10 ppm of GA can triple the size of ‘Black Finger’ berries^10^. GA is also known to potentially delay maturity, increase pedicel thickness, and increase berry abscission depending on the application time. If applied on the whole vine on sensitive varieties, GA can also harm reproductive meristems and reduce subsequent yield. The molecular aspects of the synthesis, signal perception, and transduction of GA in grapes have been reported^11,12^.

The study of the effects of cytokinins on grape development focused largely on forchlorfenuron (N-(2-chloro-4-pyridyl)-N′-phenylurea), a synthetic cytokinin known as CPPU, that has been tested and applied to regulate fruit-set, size, and shape in several fruit crops^13,14^. In grapes, CPPU was also reported to delay maturation and reduce berry skin color, increase berry pedicel thickness and rigidity, increase cuticle content, and reduce weight loss of the rachis^15–18^. CPPU is commercially applied at levels of 5 ppm or lower due to its potential adverse effects on maturation and postharvest quality. The time frame for application of CPPU is usually similar to that of GA and often in combination with GA at reduced concentrations^16^. Other cytokinins such as benzyl adenine had similar effects but a concentration of 500–1000 ppm was required to increase berry size^19^.

Phenylpropanoids are a large class of plant secondary metabolites derived from aromatic amino acids, phenylalanine in most plants or tyrosine in some monocots. The main branches of the phenylpropanoid pathway include lignans and lignins, stilbenes, coumarins, isoflavonoids, flavonoids, and PAs^20^. The biosynthesis of PAs, anthocyanins, and flavonols share common steps in the flavonoid pathway^4^. In grape berries, the first committed steps in PA biosynthesis are catalyzed by leucoanthocyanidin reductase (LAR) and anthocyanidin reductase (ANR) by converting anthocyanidins to flavan-3-ols such as (+)-catechin (C) and (−)-epicatechin (EC), respectively. The resulting (−)-epicatechin and (+)-catechin derivatives can be oxidized to quinones, which are polymerized. However, it is not clear whether this polymerization of the soluble precursors proceeds enzymatically by laccases or non-enzymatically^21^. The subunits of PA are derived from 2,3-cis-flavan-3-ols (i.e., (−)-epicatechin and (−)-epigallocatechin (EGC), as well as from 2,3-*trans*-flavan-3-ols (i.e., (+)-catechin and (+)-gallocatechin) and are most commonly linked via 4 β → 8C−C bonds^21^. The PAs are oligomeric and polymeric flavan-3-ols that can range in size from 2 to 30 or more subunits. The regulation of the phenylpropanoid pathway was studied extensively in grapes with respect to anthocyanins, PAs, and other branches of the pathway with emphasis on the role of R2R3-MYB, bHLH, and WD40 transcription factors and their target genes^4,5^.

Our previous study demonstrated that CPPU causes a marked increase in tannin content of Thompson Seedless^22^. Thompson Seedless is a major variety but it does not produce anthocyanins and is very low in volatile content. In the current study we presented the following questions: (1) how do CPPU and GA affect tannin accumulation in a variety that is rich in anthocyanins and volatile compounds; and (2) how are biological processes at ripening affected by the treatments. These questions were addressed at the phenological level, by the analysis of relevant metabolites from the phenylpropanoid pathway, by volatile composition, and by transcriptome analysis.

## Materials and methods

### Plant material and tissue collection

Experiments were carried out on *Vitis vinifera* cv. Sugrasixteen (SABLE SEEDLESS^®^) (Sun World, Bakersfield, CA) that will be referred as ‘Sable’. Vines of ‘Sable’ were grafted on Richter rootstock that was 7 year old and grown in Israel in the Lachish area (lat. 31°33′, long. 34°51′). All viticulture practices were performed as describe previously^23^. The experimental plot comprised of four replications of three vines each, arranged in a randomized block design. Clusters were manually sprayed to full wetness with the growth regulators gibberellic acid (GA_3_, ProGibb 40, Valent BioScience, Walnut Creek, CA, USA) and forchlorfenuron (CPPU, a synthetic cytokinin, SKW, Wittenberg, Germany) in a concentration of 20 and 5 ppm, respectively, with 0.025% Triton-X100 (Adama Agan, Ashdod, Israel) as a wetting agent. A combination of GA and CPPU (hereafter GA + CPPU) was also sprayed on the berry at the same concentrations. ‘Sable’ was treated on 14 May 2018 at the berry diameter of 6.0 ± 0.08 mm (E-L stage of 31)^24^. Phenological data for ‘Sable’ was collected at the time of treatment (i.e., day 0), and 7, 34, 51, and 70 days (d) post treatment (34, 51, and 70 d corresponding to stages 35, 36–37, and 38 of the modified E-L scale, respectively). Sampling for metabolites, volatiles, and gene expression was carried out at 51 and 70 d after treatment, the time points which represent the beginning and end of commercial harvest. Sampling at all time-points was of 90 berries pooled from 20 to 30 clusters randomly collected from the four vineyard replications. For metabolite and RNA-seq analysis, a disk of 14–16 mm was removed along the longitudinal axis of each berry and was frozen in liquid nitrogen. The berry disks were homogenized in three replicates of 30 disks each using an IKA homogenizer (IKA, Staufen, Germany) with liquid nitrogen and were stored at −80 °C for further analysis. The remaining part of the berries was used for measurement of total soluble solids (TSS) and titratable acidity (TA).

### Measurement of total soluble solids, titratable acidity, and tasting

Measurements of TSS and TA in ‘Sable’ berries of GA, CPPU, and GA + CPPU treatments were carried out as previously described^23^. The juice was obtained by using a fruit juicer (EF-800, Sachs, Tel Aviv, Israel) from 30 berries. TSS was determined by a digital refractometer (Atago, Tokyo, Japan) and denoted as °Brix. TA was measured by means of titration with 0.1 N NaOH to pH 8.2 with a Metrohm automatic titrator (Herisau, Switzerland) and expressed as tartaric acid equivalents. All the above analyses were performed on fresh samples at the harvest date. Informal tasting was done by three expert tasters that scored the samples in hedonic scale of 1–9 (9 being excellent taste).

### Anatomical observation of ‘Sable’ berry skin

Sample preparation and microscopic examination of ‘Sable’ berries at 33 d after GA, CPPU, and GA + CPPU treatment were performed as described by Tyagi et al.^23^. Briefly, transverse hand sections from ‘Sable’ berries of GA, CPPU, and GA + CPPU treatment were immediately immersed in a solution (v/v) containing formaldehyde, acetic acid, ethanol, and water in ratios of 10: 5: 50: 35, respectively. After fixation, tissue sections were serially diluted by ethanol and subsequently a stepwise exchange of ethanol with Histoclear (xylem substitute) was carried out. Samples were embedded in paraffin and cut with a microtome (Leica RM2245, Leica Biosystems, Nussloch, Germany) into 12 μm thick sections. Sections were stained with Safranin O that stains nuclei, lignified suberized, or cutinized cell wall in red, and with fast green FCF that stains cellulose in green-blue^25^ and sections were examined under a light microscope (Leica DMLB).

### Extraction and analysis of phenolic compounds by HPLC

Extraction and analysis of phenolics from mature berries (GA, CPPU, and GA + CPPU) of ‘Sable’ (51 and 70 d after treatment) were carried out as described previously^23^. Briefly, anthocyanins and other monomeric phenols were extracted using 0.8 g of frozen powder and homogenized in 3 mL of 50% (v/v) methanol (containing 0.1% v/v hydrochloric acid and 0.1% w/v ascorbic acid). Homogenates were mixed and incubated at 4 °C for 4 h on ice with intermittent mixing and were centrifuged at 4000*g* (Eppendorf, Hamburg, Germany) for 5 min. Pellets from the above samples were re-extracted twice and all the supernatants were pooled to make the final volume of 5 mL. Of this, 1 mL of supernatant was used for the analysis of anthocyanins and other monomeric phenols and the remaining 4 mL was stored at −20 °C. The pellet of the above extract was used further for analysis of PAs. The anthocyanins and other monomeric phenols of ‘Sable’ berries of GA, CPPU, and GA + CPPU were analyzed by Agilent Infinity series 1260 RP-HPLC (Agilent Technologies, Santa Clara, CA, USA). All data processing and analysis were accomplished using Agilent CDS ChemStation software on an Agilent 1260 Infinity HPLC (Agilent Technologies).

### PA extraction, purification, and phloroglucinol analysis

PA extraction, purification, phloroglucinolysis, and data analysis were performed as described by Tyagi et al.^23^. Briefly, the pellet from the previous section (monomeric phenolics extraction for ‘Sable’ 51 and 70 d after GA, CPPU, and GA + CPPU treatment) was vortexed in 8 mL of 70% acetone containing 0.1% ascorbic acid (w/v) and 0.05% trifluoroacetic acid (v/v) for the extraction of larger polymers. After overnight extraction at 4 °C with occasional mixing, the acetone extract was centrifuged at 4000*g* and the pellet was re-extracted twice with acidified acetone to make a final volume of 12 mL. The methanol supernatant (4 mL extract from anthocyanins and monomeric phenols) and acetone supernatant were pooled and reduced under vacuum to near dryness at 34 °C using a Buchi^®^ Rotavapor R110 (Flawil, Switzerland) and the extract was dissolved in 1 mL with methanol. PAs were purified by solid phase extraction (SPE) using bed columns containing 10 mL of Toyopearl HW-40F size-exclusion media (Sigma-Aldrich, USA) and the eluates from the SPE columns containing isolated PA were concentrated under reduced pressure, dissolved in 500 µL methanol, and stored at −80 °C until analysis. The composition of isolated PA from ‘Sable’ berries was determined by phloroglucinolysis and the reaction products were chromatographically separated by RP-HPLC on an Agilent Infinity series 1260 HPLC system (Agilent Technologies) equipped with a DAD detector using a binary solvent gradient.

### GC-MS extraction and analysis of free volatiles

Volatiles from mature berries of ‘Sable’ (51 and 70 d in GA, CPPU, and GA + CPPU after treatment) were extracted and analyzed according to the procedure described^26^. Volatiles were extracted from 2 g of frozen powder in three replicates. Volatile compounds were analyzed using MSD Chemstation E.02.00.493, and their MS description was based on the NIST library, Version 05 (Agilent Technologies, USA). Volatile compounds were identified based on the Retention Index (RI) and mass spectra, specific compounds were also identified by authentic standards as described previously^27^.

### RNA extraction, sequencing, and data analysis

Total RNA extraction and DNase treatment from 1.2 g of frozen powder of ‘Sable’ (51 and 70 d after treatment) in control (Ctrl), GA-, and CPPU-treated berries was performed as described previously^23^. RNA was sequenced by the DNA Technologies Core facility (University of California, Davis, CA, USA). Gene expression profiling was performed using a 3′-Tag-RNA-Seq protocol. Barcoded sequencing libraries were prepared using the QuantSeq FWD kit (Lexogen, Vienna, Austria) for multiplexed sequencing using both the UDI-adapter and UMI Second-Strand Synthesis modules (Lexogen). The preparation followed the recommendations of the manufacturer with two modifications, first being a reduction in fragmentation time to 10 s at 85 °C and the second was extending the reverse transcription step to 1 h at 42 °C. The fragment size distribution of the libraries was confirmed via micro-capillary gel electrophoresis on a LabChip GX system (PerkinElmer, Waltham, MA, USA). The libraries were quantified by fluorometry on a Qubit fluorometer (Life Technologies, Carlsbad, CA, USA), and combined in equimolar ratios. The library pool was quantified via qPCR with a Kapa Library Quant kit (Kapa Biosystems/Roche, Basel, Switzerland) on a QuantStudio 5 system (Applied Biosystems, Foster City, CA, USA). Up to 48 libraries were sequenced per lane on a HiSeq 4000 sequencer (Illumina, San Diego, CA, USA) with single-end 100 bp reads.

The raw reads of 18 libraries—three biological replications for each of the three treatments, Ctrl, GA, and CPPU at 51 or 70 d—were subjected to quality filtering and adapter removal by HTStream software (https://github.com/s4hts/HTStream). Cleaned sequences were mapped to the genome version Vcost.3 of *V. vinifera* downloaded from the site (https://urgi.versailles.inra.fr/Species/Vitis/Data-Sequences). STAR software was used to align clean reads to the reference genome. Sequence data was deposited to BioProject at NCBI under the accession PRJNA686849. Bioconductor DESeq2 was used to identify differentially expressed transcripts, based on the count estimates for each transcript. Transcripts were regarded as differentially expressed if they reached the threshold of FDR < 0.05 and the log2-fold change was < −1 or >1. GO enrichment analysis was done using Blast2Go. KEGG pathway enrichment was done using the KOBAS 3.0 server (http://kobas.cbi.pku.edu.cn/). The expression data was normalized by using the trimmed mean of M values method (TMM). For the clustering we used cutree in R that takes as parameters *k* number and cut the hierarchical clustering tree into *k* clusters (https://www.rdocumentation.org/packages/stats/versions/3.6.2/topics/cutree).

### Statistical analysis and data representation

A minimum of three biological replicates were used for each experiment and all results were expressed as mean ± SD (standard deviation) or as specified in the figure legends. Each biological replicate consisted of 30 berries. Statistical analysis was done by ANOVA followed by Tukey post-hoc tests or Student *t*-tests as indicated by JMP (ver. 13.0; SAS Institute, Cary, NC, USA). Principal component analysis (PCA) and variables importance in projection (VIP) score were performed to obtain overall clustering of the samples from the volatile analysis, using Metaboanalyst 4.0^28^. Venn diagrams were performed by the free online BEG tool (http://bioinformatics.psb.ugent.be/webtools/Venn/). Figures were prepared with R packages ggplot2, tidyverse, agricolae, svDialogs, and multcompView.

## Results

### Effect of early GA and CPPU treatment on ‘Sable’ berry properties along development

**Fig. 1:**
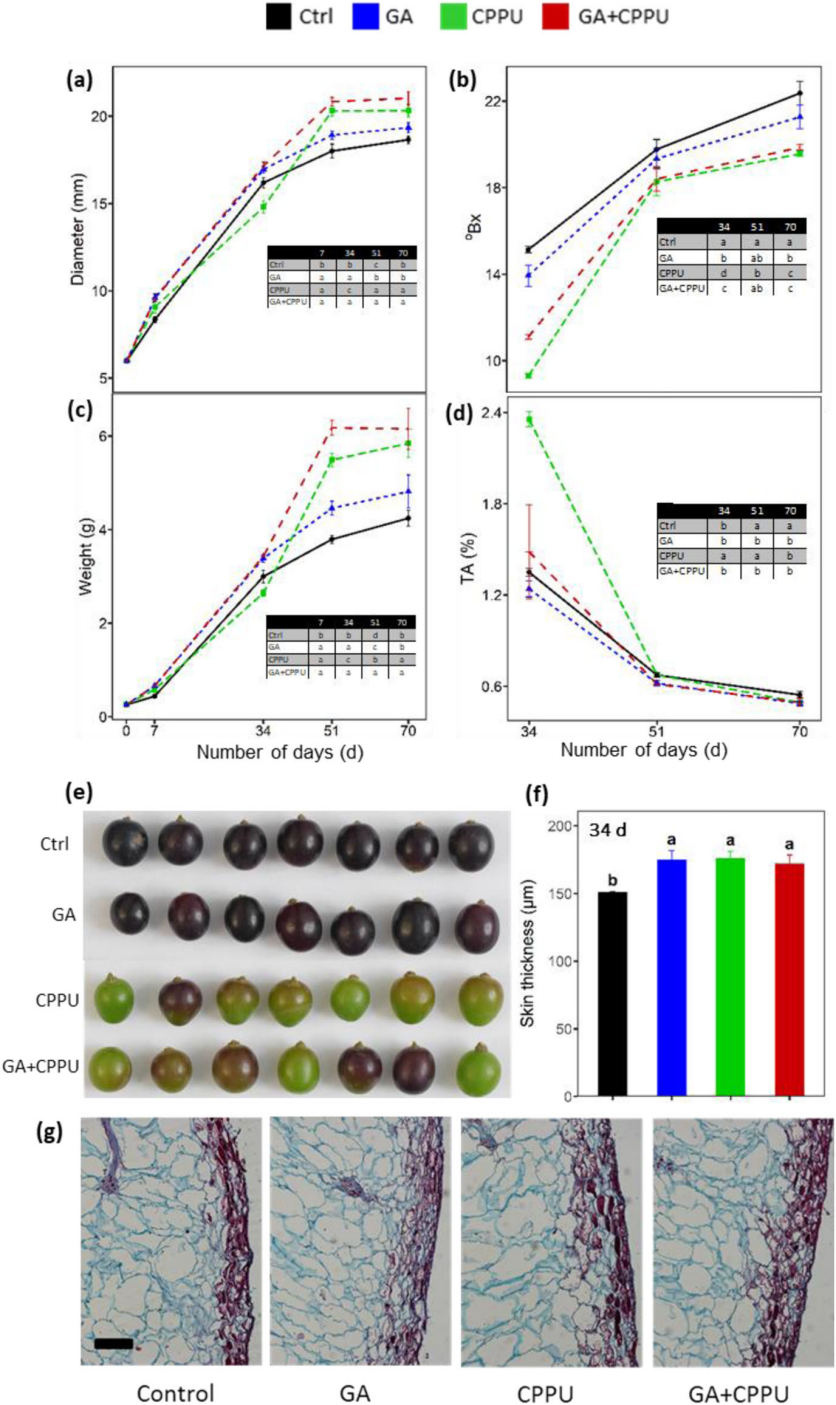
Effect of growth regulator treatments after berry set on berry properties during development and ripening in ‘Sable’. Berries were treated with GA, CPPU, and GA + CPPU after the berry set. **a** Diameter (mm), **b** °Brix, **c** weight (g), **d** titratable acidity (% TA), **e** berry appearance 34 d after treatment, **f** berry skin thickness (µm), and **g** representative berry sections stained with Safranin, from the Ctrl, GA, CPPU, and GA + CPPU 34 d after treatments. (**a**–**d**) Sample size was of 30 berries and values are the mean and standard deviation of three replications and the table in the panel indicates if the differences among treatment are significant by Tukey post-hoc test at *p* ≤ 0.05. Value in panel (**f**) are mean and standard deviation of 9 sections from 3 berries and different letters above each bar denote significant differences between the treatments. The horizontal black bar represents a size of 100 µm (**g**)

Gibberellin and cytokinin applied at fruit-set are known to increase berry size, but due to the complexity of agricultural systems, the intensity of the response can vary among seasons. The PGR treatments were performed at a fruitlet size of 6.0 ± 0.08 mm on ‘Sable Seedless’. Fruitlet diameter and weight increased significantly by application of both GA and CPPU measured 7 d after treatment (Fig. 1a, c). Interestingly, at 34 d post treatment, berry diameter and weight of CPPU-treated berries was smaller than the control but this trend was reversed at later time points. At 51 and 70 d after the treatments, CPPU increased berry weight and diameter significantly with respect to the control and the GA treatment. The GA treatment increased berry size by 17.5% and 13.4% at 51 and 70 d, respectively, but at a later time point, the difference was not statistically significant from the control. The diameter and weight of berries treated with the combined treatment of GA + CPPU was higher than that of CPPU alone at both 51 and 70 d. Brix of the control grapes was higher at all time-points followed by GA and CPPU (Fig. 1b). At 34 d, berries treated with GA + CPPU had higher Brix as compared to CPPU-treated berries, but at later time points there were no differences among the two treatments. CPPU had a major effect on TA that was much higher than the other treatments at 34 d after treatment (Fig. 1d). At both 34 and 51 d, GA seemed to mitigate the effect of CPPU on TA. With respect to appearance of the berries 34 d after the treatment, there was a clear delay in color development by CPPU and the treatment of GA + CPPU (Fig. 1e). Transverse sections of the berries showed that the epidermal layer was thicker either in both GA- and CPPU-treated berries or the combined treatment as compared to the untreated control (Fig. 1f, g). Informal tasting done by experts indicated excellent taste rated as 8 with multiple aromatic notes for the control and GA treatments, while in berries from the CPPU treatment there was some astringency that reduced the score to 7.5 or 7.0 for the combined treatment.

### CPPU but not GA treatments alter the levels of flavonoid compounds

**Fig. 2:**
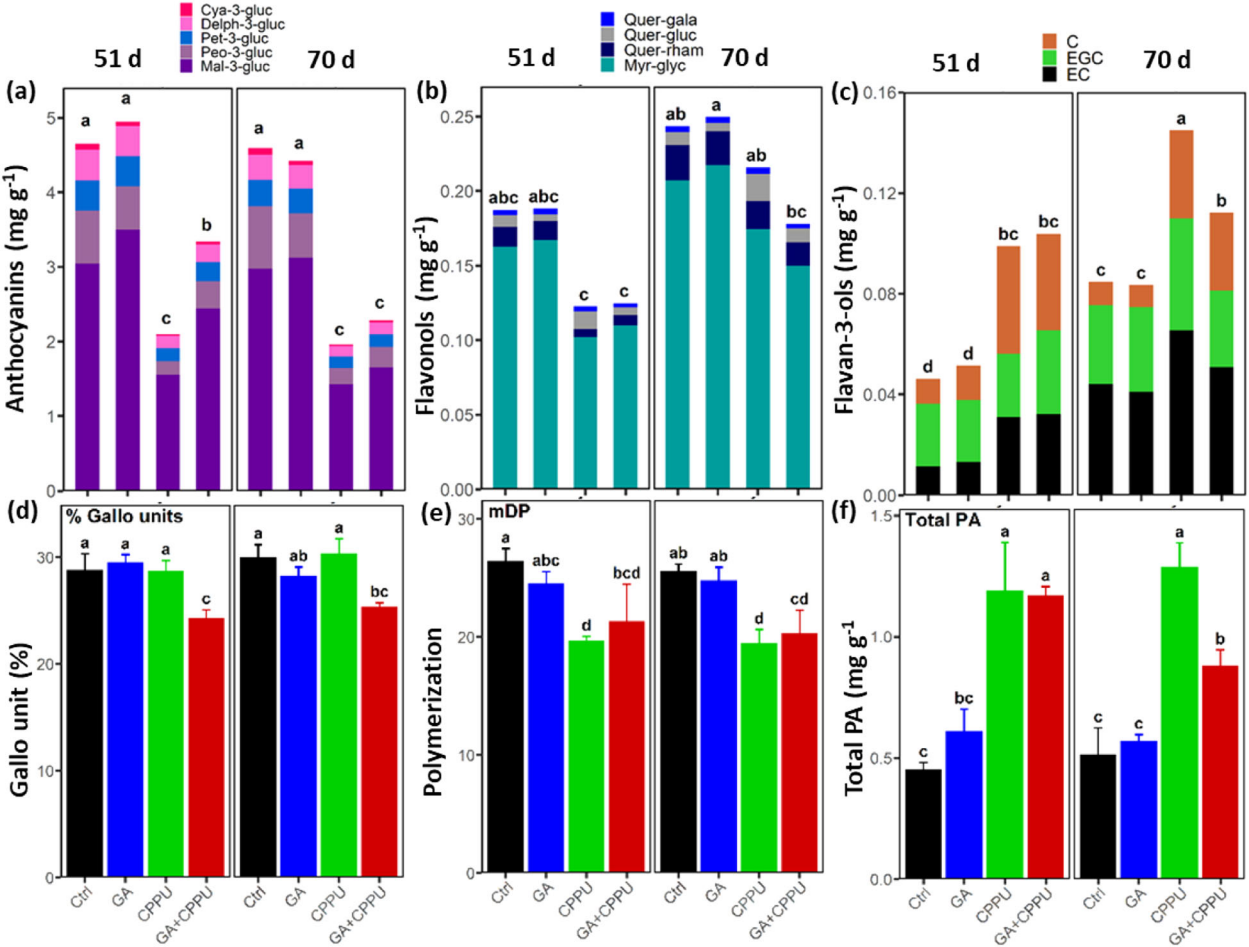
Polyphenol monomer and proanthocyanidin (PA) content in ‘Sable’ at 51 and 70 d after growth regulator treatments. **a** Total anthocyanins (glucoside forms), **b** flavonols, **c** flavan-3-ols, **d** gallo unit, **e** mean degree of polymerization (mDP), and **f** total PAs were measured in berries treated with GA, CPPU, and GA + CPPU as compared to the control. Separation and analysis of the monomers and PAs were done by RP-HPLC. Amounts of PAs were the sum of all monomers and analyzed after phloroglucinolysis on RP-HPLC. Value of monomers and PAs are expressed as mg g^−1^ FW (Fresh Weight) and are the mean and standard deviation of three replications. Gallo units (%) is percentage of galloyl units (EGC-P and EGC (epigallocatechin)) of the total. Different letters above each bar denote significant differences between the treatments by Tukey post-hoc test at *p* ≤ 0.05

The cytokinin CPPU was shown to increase total tannins in Thompson Seedless that bears green berries^22^. It was therefore of interest to determine what effect CPPU has on black berries and if GA has similar effects. HPLC analysis was carried out for glucosides, acylated and coumaroylated forms of delphinidin, cyanidin, petunidin, peonidin, and malvidin (Fig. 2a and Table S1). CPPU and GA + CPPU treatments reduced the levels of the majority of these compounds at both 51 and 70 d as compared to the control. Quantitatively, CPPU reduced the levels of the anthocyanin glucosides by ca. 50% at both time points. The GA + CPPU treatment reduced the levels of the anthocyanin glucosides to ca. 75% and 53% at 51 and 70 d, respectively, as compared to the control. At 51 d post treatment, GA reduced the inhibitory effect of CPPU but this effect was not maintained at 70 d (Fig. 2a). Anthocyanin glucosides are the major form present in ‘Sable’ comprising 63–71% of the total anthocyanins and the remaining are acetylated and coumaroylated forms (Table 1). Interestingly, CPPU reduced the proportion of the glucoside forms at both 51 and 70 d and this decrease was accompanied by an increase in coumaroyl glucosides at both time points and acetylated glucosides at 70 d. GA had an intermediate effect on the proportion of the anthocyanin forms.

**Table 1:** Influence of GA, CPPU, and GA + CPPU treatments on ‘Sable’ proportion (%) of glucoside, acetylated and coumaroylated anthocyanin forms at 51 and 70 d after treatment

Anthocyanin forms	51 d				70 d			
	Ctrl	GA	CPPU	GA + CPPU	Ctrl	GA	CPPU	GA + CPPU
Gluc	71.8 ± 0.17	***69*** ± ***0.74***	***67.9*** ± ***0.45***	***67.7*** ± ***0.12***	68.7 ± 0.48	66.9 ± 0.32	***63.4*** ± ***0.18***	***64.7*** ± ***0.49***
Acetylgluc	8.2 ± 0.03	8.4 ± 0.15	***8.3*** ± ***0.05***	8.4 ± 0.08	7.5 ± 0.1	***8.4*** ± ***0.14***	***9.3*** ± ***0.18***	***9*** ± ***0.07***
Coumgluc	20 ± 0.16	***22.6*** ± ***0.59***	***23.8*** ± ***0.41***	***23.9*** ± ***0.04***	23.7 ± 0.45	24.7 ± 0.2	***27.4*** ± ***0.31***	***26.3*** ± ***0.43***

Different anthocyanin form are anthocyanin-3-*O*-glucoside (Gluc), anthocyanin-3-*O*-acetylglucoside (Acetylgluc), and anthocyanin- 3-*O*-(6-*p*-coumaroyl) glucoside (Coumgluc). Values in bold and italic differ significantly from the control according to Student *t*-test at *p* ≤ 0.05 and *n* = 3

Flavonols are an important branch of the flavonoid pathway and therefore it was of interest to determine the effect of the treatments on major grape flavonols (Fig. 2b). Myrcetin glycoside was the major flavonol detected with an average of 81–89% among the compounds tested. While variation among replications was significant, two trends are worth noting. At 51 d, CPPU alone or in combination with GA reduced the levels of flavonols to ca. 60% of the control. At 70 d there was an increase in levels of the flavonols and decrease in the effect of CPPU. Quercetin galactoside did not significantly differ among the treatments.

Flavan-3-ols are the building blocks of the PA chains that are synthesized in the early stages of berry development. Figure 2c displays the three major flavan-3-ols: (+)-catechin, (−)-epicatechin (EC), and (−)-epigallocatechin (EGC). Clearly, CPPU-treated berries contained more flavan-3-ols and their level increased during ripening. At the late sampling, GA + CPPU reduced the level of the flavan-3-ols as compared to the CPPU treatment alone. The hydroxycinnamic acid derivatives, caffeic acid, caftaric acid, coutaric acid, and ferulic acid changed in different ways: caftaric acid was highest in the early stage in CPPU-treated berries; caffeic acid levels were reduced by CPPU at the early stage and it was absent in the late harvest; coutaric acid levels were induced by CPPU and reduced by the combined treatment relative to the control; the levels of ferulic acid were low but increased with ripening without a distinct pattern (Table S1).

### CPPU increases PA levels but shortens the polymer length in berries

To further investigate the effect of GA, CPPU, and GA + CPPU on PA composition, phloroglucinolysis was performed and differences were monitored using HPLC (Figs. 2d–f, S1, and Table S2). Phloroglucinolysis hydrolyzes the polymeric PAs generating terminal units (C, EC, and ECG (epicatechin gallate)) and extension units with phloroglucinol adduct (labeled by –P). CPPU increased the levels of C, EC, EC-P, and EGC-P. GA did not affect the levels of the PA monomers while the treatment with GA + CPPU reduced the levels of C, ECG, EC-P, and EGC-P only at 70 d after the treatments (Fig. S1). Monomeric PAs did not change with ripening with the exception of ECG that was lower at 70 d compared to the control treatment. The total PA level clearly shows the effect of CPPU and also shows that the combined treatment reduced the level of PAs at 70 d in agreement with the data on the free monomers (Fig. 2c). The treatments with GA or CPPU had minor effects on the % galloyl units but the combined treatment reduced their level. The mean degree of polymerization (mDP) was significantly lower in CPPU and GA + CPPU treatments as compared to the control (ca. 26 mDP in the control versus ca. 20 mDP in the treatments).

### Volatile compounds affected by the growth regulator treatments

**Fig. 3:**
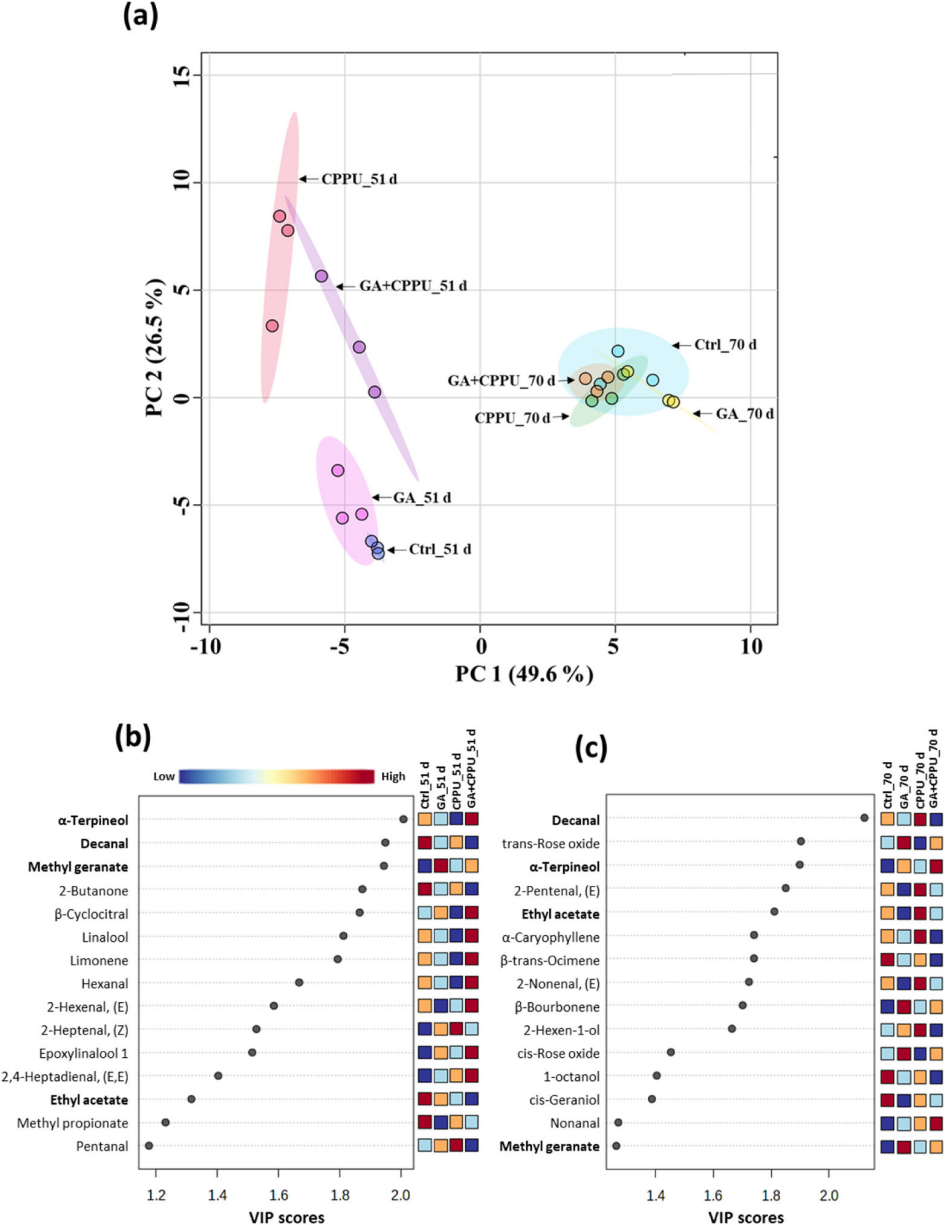
Volatile content ‘Sable’ at 51 and 70 d after growth regulator treatments. Volatile analysis of samples from GA-, CPPU-, and GA + CPPU-treated berries compared to the control was done by headspace SPME GC-MS analysis. **a** Principal component analysis (PCA) of volatile compounds was constructed for all treatments and time points. Variable importance in projection (VIP) scores of the variables at 51 d (**b**) and 70 d (**c**) for control, GA, CPPU, and GA + CPPU. The VIP score of a variable is calculated as a weighted sum of the squared correlations between the PLS-DA components and the original variable. Name in bold letters denotes common compounds (**b**) and (**c**). Color scale in (**b**) is applicable to (**c**) also

To evaluate the effect of GA, CPPU, and GA + CPPU treatment at berry set on the volatiles composition, berries of ‘Sable’ were sampled 51 and 70 d after the treatments. GC-MS analysis allowed identification of 58 (51 d) and 67 (70 d) volatile compounds with quantitative data calculated relative to the internal standard (Table S3). Analysis of the differences in volatile content by ANOVA showed that 62 of the 68 compounds present at both time points differed in content by treatments or time (Table S4). PCA indicated strong separation of the volatile compounds according to the harvest time with PC1 at 49.6% (Fig. 3a). PC2 contributed 26.5% of the total variance and reflected the effect of CPPU at 51 d, but at 70 d there was convergence of the variance and overall differences among the treatments seemed insignificant. VIP score analysis for the volatile compounds at 51 d pointed to 15 compounds having significant scores (Fig. 3b). CPPU suppressed the levels of the monoterpenes, α-terpineol, linalool, and limonene, as well as the carotenoid derivative, β-cyclocitral and the fatty acid derivative, hexanal. However, GA + CPPU increased the levels of these compounds. The levels of 2-hexenal (Z) and pentanal were higher in CPPU-treated berries while GA increased the level of methyl geranate. At 70 d, CPPU increased and GA or GA + CPPU decreased the levels of decanal and 5 other compounds (Fig. 3c). In contrast, GA increased the levels of *trans* and *cis*-rose oxide, β-bourbonene, and methyl geranate. Decanal, methyl geranate, ethyl acetate, and α-terpineol were common among the two time points and the first two compounds showed the same pattern.

### CPPU but not GA had long-term effects on gene expression in berries

**Fig. 4:**
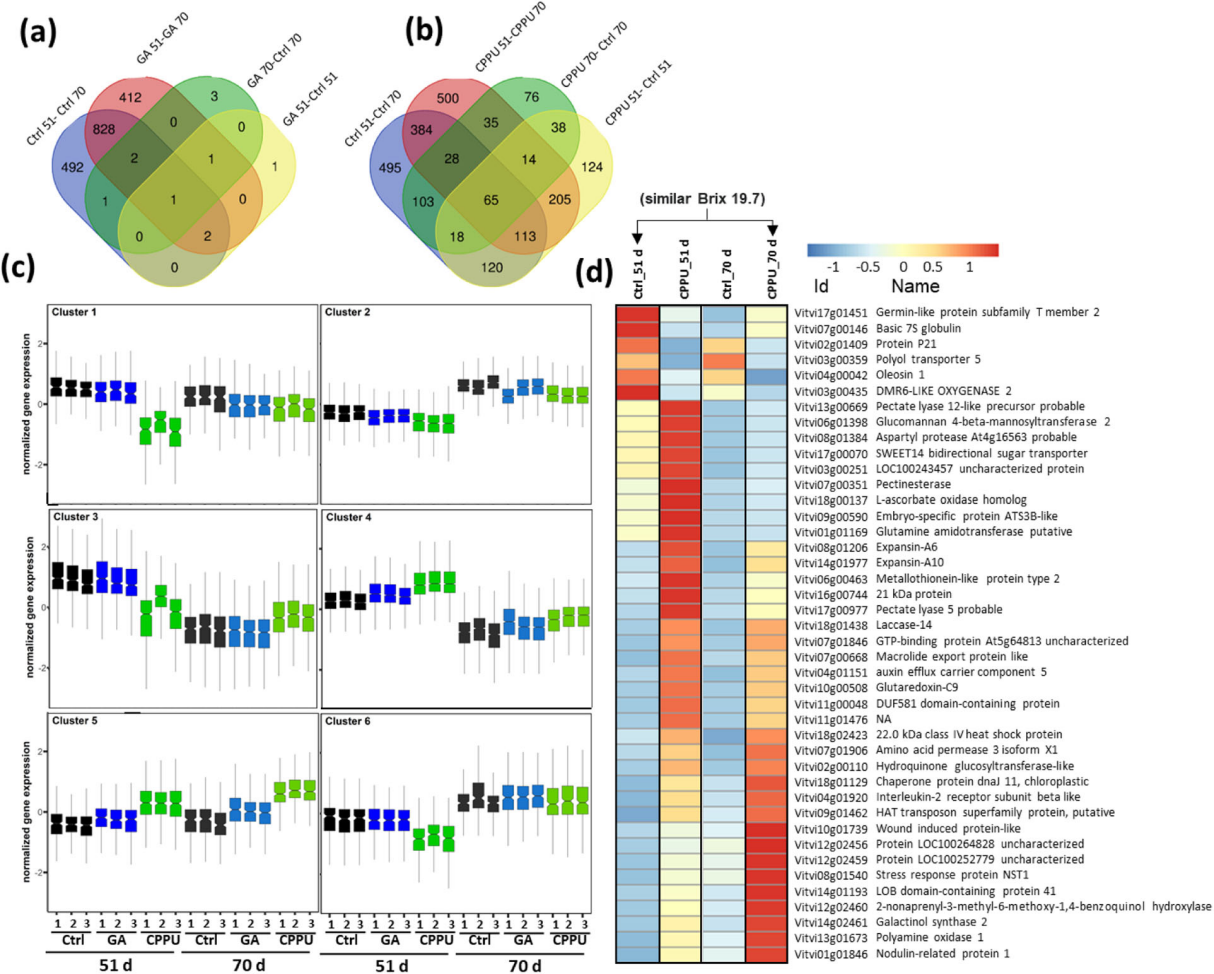
RNA-seq analysis of differential expressed genes in ‘Sable’ at 51 and 70 d after growth regulator treatments. **a** Venn diagram shows differentially expressed genes (DEGs) in the combinations of treatments and time points: control – Ctrl; gibberellin - GA; 51 d – 51; 70 d – 70. **b** Venn diagram between the control and CPPU treatments at 51 and 70 d. **c** DEGs were clustered at threshold of log2 ≥ 1 or ≤ −1 and *p*_adj_ value ≤ 0.05, and the 6 main clusters are presented. **d** Heat map of DEGs common among control and CPPU at both in 51 and 70 d, and at similar Brix (control at 51 d and CPPU at 70 d)

PGRs may have multiple immediate effects on gene expression, but, in this study, we were interested in understanding the long-term effects of GA and CPPU on the berries at harvest. To this end, RNA-seq was carried out on ‘Sable’ berries at 51 and 70 d after the treatments, the time points that can be defined as early and late ripening with respect to the untreated control. A total of 3801 differentially expressed genes (DEGs) among all treatments were identified (Table S5). Venn diagram analysis of control vs. GA showed that there were hardly any differences between the control and GA treatments either at the early or late ripening, but there were significant differences in gene expression among the ripening stages (Fig. 4a). In contrast, the CPPU treatment imposed significant differences on gene expression at either the early or late stages of ripening (Fig. 4b). Six main clusters representing gene expression patterns were identified (Fig. 4c). KEGG pathway enrichment showed that only cluster 3 and cluster 4 contained significantly enriched pathways (Table 2). Six out of the 13 processes related to cluster 3 were assigned to the phenylpropanoid pathway. The first process registered for cluster 3 was ‘circadian rhythms’ but analysis of the relevant KEGG map suggested that all the 20 enriched genes in this bin were actually family members of *chalcone synthase* (*CHS*) which is a central flavonoid pathway. The ‘ubiquinone and other terpenoid-quinone biosynthesis’ bin included *4CL*, *trans-cinnamate 4-monooxygenase*, and *isochorismate synthase*. Other bins of cluster 3, excluding the large unspecific bins of ‘Biosynthesis of secondary metabolites’ and ‘Metabolic pathways’, will be discussed later. Cluster 4 contained 2 out of 5 bins assigned to ‘photosynthesis’ or ‘photosynthesis – antenna proteins’. The genes in these bins belonged to photosystem I, photosystem II, cytochrome b6/F complex, and LHC1 and LHC2 complexes. In the ‘Steroid biosynthesis’ bin, genes included *squalene synthase*, *squalene monoxygenase, a*nd other sterol biosynthesis genes upstream of brassinosteroid biosynthesis.

**Table 2:** Process enrichment by KEGG analysis for DEGs belonging to cluster 3, cluster 4, or DEGs among early control and late CPPU treatment

KEGG Term	KEGG ID		All DEGs		Control 51 d /CPPU 70 d^f^	
		Background^c^	Input^d^	*p*-Value^e^	Input	*p*-Value
Cluster 3						
Circadian rhythm - plant	vvi04712	72	20	8.01E-14	25	1.79E-19
Flavonoid biosynthesis	vvi00941	110	23	8.01E-14	28	4.16E-19
Biosynthesis of secondary metabolites	vvi01110	1271	69	1.38E-11	69	4.97E-12
Metabolic pathways	vvi01100	2390	88	1.32E-06	87	9.65E-07
Phenylpropanoid biosynthesis	vvi00940	202	18	6.89E-06	16	6.54E-05
Phenylalanine metabolism	vvi00360	55	9	5.43E-05	8	0.000237
Biosynthesis of secondary metabolites^a^	vvi00999	15	5	0.000359	5	0.000237
Stilbenoid, diarylheptanoid and gingerol^b^	vvi00945	39	6	0.001958	9	3.74E-06
MAPK signaling pathway - plant	vvi04016	142	11	0.001958	10	0.004328
Ubiquinone and other terpenoid-quinone^b^	vvi00130	43	6	0.002773	—	—
Phenylalanine, tyrosine and tryptophan^b^	vvi00400	50	6	0.005204	—	—
Plant–pathogen interaction	vvi04626	236	13	0.008895	—	—
Plant hormone signal transduction	vvi04075	291	13	0.043811	13	0.034004
Cluster 4						
Photosynthesis	vvi00195	21	21	2.65E-05		—
Metabolic pathways	vvi01100	192	192	0.000653		—
Biosynthesis of secondary metabolites	vvi01110	113	113	0.000744		—
Photosynthesis - antenna proteins	vvi00196	7	7	0.010617		—
Steroid biosynthesis	vvi00100	9	9	0.017040		—

Input/background number refer to the number of genes enriched for a specific process among all genes belonging to this process

^a^Unclassified

^b^Biosyntheiss

^c^Number of the genes in the term

^d^Number of genes enriched

^e^Corrected *p*-value

^f^Similar Brix group

### Comparison of the effects of CPPU on gene expression at similar Brix

Because GA did not contribute a significant number of genes to the DEG pool, it was eliminated from further analysis. There were major differences in ripening between the untreated grapes and grapes treated with CPPU, and this was also expressed in the number of DEGs at both early and late sampling. At the early and late sampling, there were 697 and 382 DEGs between the untreated and the CPPU-treated berries, respectively, and the reduction in the number of DEGs was mainly in the late sampling for genes with lower expression following CPPU treatment (Tables S6 and S7). KEGG analysis of these two groups did not show any functional bin enrichment. In a separate analysis, the treatments were compared on the basis of similar Brix; this comparison was possible because the untreated berries reached a Brix of 19.8 at 51 d while the CPPU-treated berries reached Brix of 19.6 at 70 d. Comparing these two groups resulted in 313 and 589 DEGs with higher and lower expression from the untreated and CPPU-treated berries, respectively (Table S8). KEGG analysis demonstrated that in this ‘similar Brix’ group there was enrichment of 11 functional bins similar to those already present in cluster 3 (Table 2).

A subset of 42 genes showed significant differences in three combinations: (1) Control vs. CPPU at 51 d; (2) Control vs. CPPU at 70 d; (3) Control at 51 d vs. CPPU at 70 d (similar Brix) (Fig. 4d). Interestingly, very few DEGs had higher expression in the untreated berries at sampling of 51 and 70 d. These genes were a *germin-like protein*, basic *7* *S globulin* (similar to *xylanase inhibitor* in Arabidopsis), protein P21 (similar to osmotin-like protein in Arabidopsis), *polyol transporter 5* (similar to SIMPT4 in tomato expressed in late stages of fruit development), *oleosin 1*, and *DMR6-like oxygenase 2* that functions in response to pathogens. The DEGs that had higher expression in response to CPPU could be assigned mainly to cell wall-related and stress-related functions. The cell wall-related DEGs included, in part, *expansins*, *pectinesterase*, *pectate lyases*, and *NST1* that is considered a regulator of secondary cell wall biosynthesis^29^. The stress-related DEGs included the chaperone *dnaJ 11*, *Nodulin-related protein 1*, a *metallothionein-like protein*, and the hypoxia-associated genes *LOB domain-containing protein 41* and a *2-nonaprenyl-3-methyl-6-methoxy-1,4-benzoquinol hydroxylase*.

**Fig. 5:**
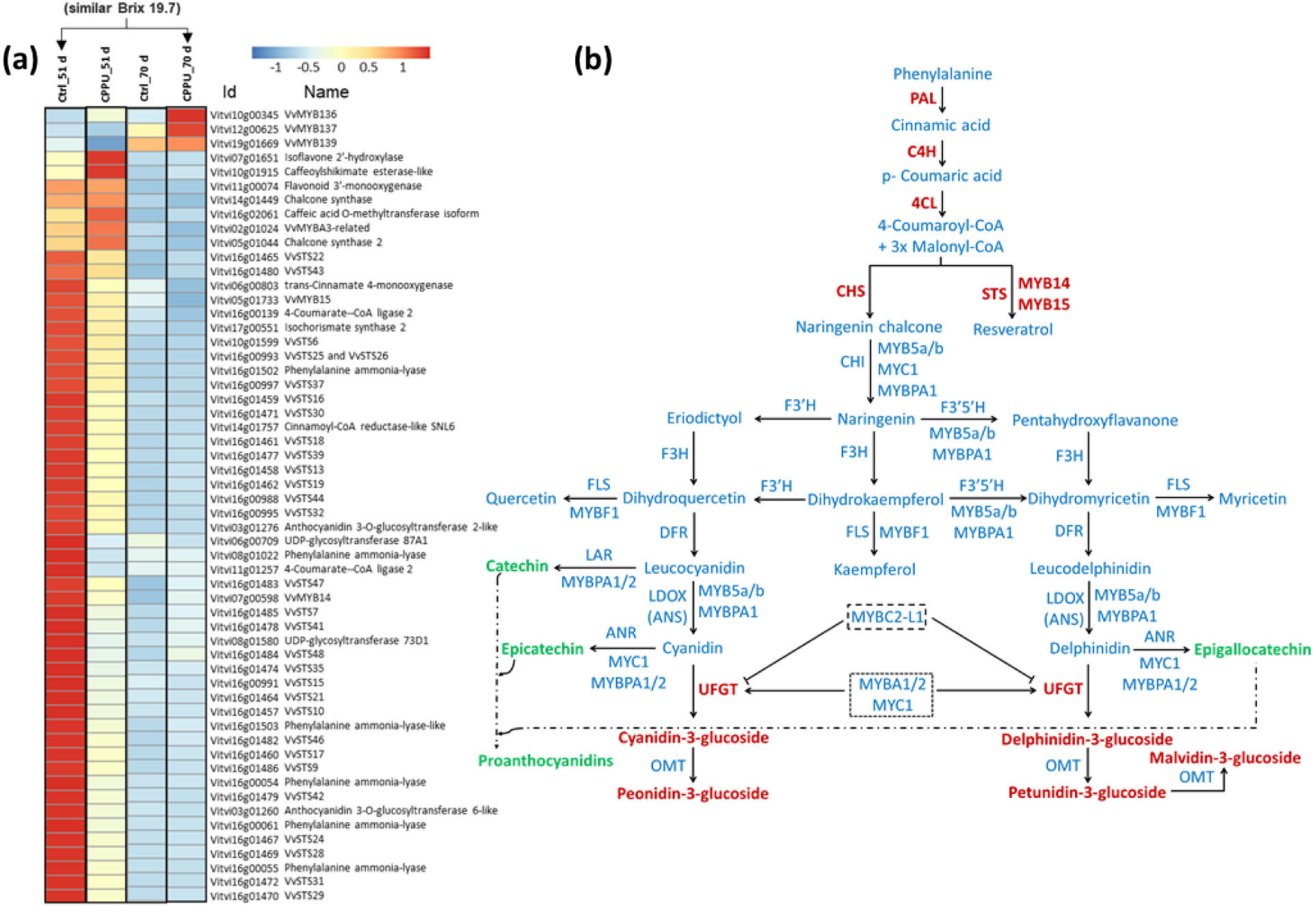
Expression of phenylpropanoid-related genes and their position on the phenylpropanoid pathway. **a** Heat map of phenylpropanoid-related DEGs among control and CPPU treatment at 51 d, control and CPPU at 70 d, and control at 51 d and CPPU at 70 d having similar °Brix (indicated by arrow). **b** A scheme of the phenylpropanoid pathway with metabolites and genes affected by the CPPU treatment. The metabolites and genes in blue letters on pathway are not detected or remain unchanged. Green or red colored letters denote metabolites or gene up-regulated and down-regulated, respectively. PAL, Phenylalanine ammonia-lyase; C4H, cinnamate 4-hydroxylase; 4CL, 4-Coumarate-CoA ligase; CHS, chalcone synthase; CHI, chalcone isomerase; F3H, flavanone 3-hydroxylase; F3′H, flavonoid 3′-hydroxylase; F3′5′H, flavonoid 3′5′-hydroxylase; DFR, dihydroflavonol 4-reductase; LDOX, leucoanthocyanidin dioxygenase; UFGT, UDP glucose:flavonoid 3-*O*-glucosyltransferase; OMT, O-methyltransferase; FLS, flavonol synthase; LAR, leucoanthocyanidin reductase; ANR, anthocyanidin reductase. Panel (**b**) was modified from Kuhn et al.^4^

The phenylpropanoid-related KEGG bins included *PAL*, *trans-cinnamate 4monooxygenase*, *4CL*, *caffeic acid O-methyltransferase*, and *cationic peroxidase* 1. The flavonoid bin included *chalcone synthase* (CHS) and the stilbenoid bin included *stilbene synthases* (STSs). The expression pattern of 56 DEGs related to the phenylpropanoid pathway that are significantly different among early control and late CPPU treatments (similar Brix group) is displayed in Fig. 5a. Interestingly, among the regulatory MYB genes ‘*MYB14, MYB15*, and *MYBA3*’ had lower expression following CPPU treatment, while three MYB genes ‘*MYB136, MYB137*, and *MYB139*’ had higher expression in CPPU-treated at similar sugar levels. Of the WRKY family, 2 genes (*WRKY22* and *WRKY49*) had higher expression while 5 genes (*WRKY05, WRKY24, WRKY40, WRKY43*, and *WRKY51*) had lower expression in CPPU-treated berries (Table S8). Of the structural genes *PAL*, *C4H*, *4CL*, *CHS*, *UFGT*, and remarkably, 28 STS genes, had lower expression in CPPU-treated berries (Fig. 5a). A scheme of the phenylpropanoid pathway with metabolites and genes affected by the CPPU treatment is shown in Fig. 5b.

The ‘MAPK signaling’ bin largely overlapped the ‘Plant hormone signal transduction’ bin. This hormone-related KEGG bin was enriched for the following genes: Auxin, *AUX/IAA* and *SAUR*; Cytokinin, A*-ARR*; Gibberellin, *GID2*; ABA, *PYR/PYL* and *PP2C*; Ethylene, *ETR* and *ERF*1/2; Brassinosteroids, *BAK1* and *TCH4;* and Jasmonic acid, *JAZ* (Fig. S2). Other enriched genes in the MAPK signaling included *WRKY33*, *MKK9*, and *CaM4*.

## Discussion

Gibberellin has been used to enlarge seedless grape berries for many years^9^. Some cultivars are very sensitive to the treatment while others have a modest response^10^. Sable Seedless can be assigned to the category of ‘modest response to GA’, with a significant effect on size at early sampling that is reduced at late harvest. The transcriptomic data for both 51 and 70 d after the treatment, corresponding to early and late commercial harvest, suggest that GA did not have long-term effects on biological processes and this was also confirmed in the profile of phenylpropanoid metabolites. However, GA did delay maturity as expressed by lower TSS and TA (Fig. 1b, d), and also had an effect on volatile compounds (Table S3). In addition, when combined with CPPU, GA had some additive effects on fruit weight but it mitigated the response to CPPU with respect to acidity and reduction in some phenylpropanoid metabolites (Figs. 1d and 2). It is likely that higher doses of GA than the concentration used in this study (20 mg L^−1^), but still considered in the range of commercial practice for this cultivar, or multiple applications, would further increase the size and delay the ripening as previously reported in many studies^30^. Our data and the literature support the notion that GA can trigger quantitative effects on berry size, but does not have a major impact on ripening processes under standard practice.

In contrast to GA, CPPU affected a wide range of phenotypic, biochemical, and molecular responses. Since the introduction of CPPU, there have been a number of studies in grapes reporting beneficial effects on fruit size, and delays in maturity, abscission, rachis browning, decay, cuticle formation, volatile profile and gene expression^8,15–18,31^. Our study on the colorless cultivar ‘Thompson Seedless’ pointed to the effect of CPPU on total tannin content resulting in higher astringency^22^. It was therefore of interest to study how the interplay between the anthocyanin branch and the PA branch of the pathway is manifested in colored cultivars. Our data clearly shows that synthesis of PA is at the expense of anthocyanins with no particular effects on the type of anthocyanins produced (Fig. 2a and Table S1). The fact that the level of flavonols was also reduced points to the possibility that either PA synthesis was induced by CPPU or that there was multiple repression of both the flavonol and anthocyanidin branches by genes such as *MybA1* and *MybF1*^32^. One interesting trend was the increase of (−)-epicatechin during late ripening, both in the control and CPPU-treated berries (Fig. 2c), in contrast to previous results showing that flavan-3-ols in the skins peak at 2–3 weeks after veraison^33^. This increase can result from degradation of PAs, but there was no evidence for this in the total PA levels and the only PA monomer that decreased in content during ripening was ECG (Fig. S1). Both flavan-3-ols, (+)-catechin and (−)-epicatechin, had higher concentration in CPPU-treated grapes and, thus, it can be hypothesized that there was a concomitant induction of their biosynthetic genes, *LAR* and *ANR* by TFs such as *MybPA1* and *MybPA2*^34,35^. CPPU did not affect the expression level of *VvMybPA1* at 51 d but the level of this gene was reduced at 70 d post treatment (Table S7). It is not known if the gene was actually induced by CPPU before veraison. It should however be noted that PAs are synthesized prior to veraison while anthocyanins are synthesized during or after veraison, bringing up the possibility that the decrease in anthocyanins may be due to deficiency in substrates in the berry rather than repression of the rate-limiting genes. In previous studies, cytokinins were shown to promote anthocyanin synthesis in shoots and leaves of *Arabidopsis* with concomitant transcriptional activation of the pathway^36^. Likewise, introduction of a bacterial *ipt* gene to tobacco leaves increased the level of phenolic compounds^37^.

The inherent discrepancy of comparing gene expression by treatments that delay maturity was partially compensated by comparing differential expression of phenylpropanoid-related genes at similar Brix, i.e., control at 51 d and CPPU at 70 d after treatment (Fig. 4d). There is a remarkable reduced expression of 28 STSs in CPPU-treated berries (Fig. 5a). The STS multigene family in grapes includes 48 annotated genes^38^. Expression maps of STS genes suggest induction of gene expression by multiple biotic stresses including ethylene and jasmonate, while abiotic stresses had both suppressive and inductive effects^39^. Therefore, according to our data cytokinin may contribute to long-term inhibition of Vv*STS* expression. Of the upstream genes in the phenylpropanoid pathway, 6 DEGs corresponded to *PAL* pointing to a trend of overall reduction in the pathway during ripening. Other genes included *C4H, 4CL*, *CHS*, *UFGT* (2 DEGs each), and 9 other structural genes of the pathway. Of the related transcription factors, *MYB14* and *MYB15* are considered as activators of *STS* gene expression^39,40^ and their low level is in agreement with the low expression of the *STS* genes. The expression of *MYB14* and *MYB15* was shown to increase with ripening in Pinot Noir grapes^41^, and therefore delay in ripening mediated by CPPU may reduce their expression. *MybA3* was shown to be a truncated non-functional protein^42^. *MYB136, MYB137*, and *MYB139* showed high expression in CPPU-treated berries, and it will be interesting to find out if they are targets for cytokinin signaling in plants and bona fide regulators of Vv*STS* genes. Of the WRKY genes, *WRKY24* and *WRKY43* are reported to be co-expressed with STS and MYB genes^39^. For example, *MYB14* and *MYB15* were co-expressed with *STS21, STS29, STS41*, and *STS48* in agreement with our data (Fig. 5a). Like *MYB14* and *MYB15, WRKY24* alone was shown to interact directly with the promoter of *STS29*.

A previous study on the effect of CPPU in ‘Shine Muscat’ grapes focused on transcriptome changes 60 d after the treatment and volatile profile changes 80 d after the treatment (i.e., when the berries reached very high maturity)^31^. Similar to the previous report, our data showed that some genes of the phenylpropanoid pathway, e.g., *PAL*, *4CL*, and *UFGT* had lower expression following CPPU treatment as well as fatty acid biosynthesis genes, amino acid biosynthesis, and volatile-related processes. Higher expression was observed for carotenoid-related genes, fatty acid metabolism genes, and auxin-related genes. In hormonal signaling, Wang et al.^31^ identified three accessions for *TIFY 5A*, two of which had higher expression at CPPU application level of 5 mg L^−1^ but with reduced expression at a level of 10 mg L^−1^ CPPU. This is consistent with our study where one of the *TIFY 5A* had increased expression at both 51 and 70 d (Tables S6 and S7). Another set of affected hormone-signaling genes in our study was that of the cytokinin receptor genes, of which *ARR-5* was repressed by CPPU at the late stage (Table S7). In contrast, in ‘Shine Muscat’ these genes were induced by CPPU. With respect to the volatile profile, similar reduction in terpenoids was encountered following CPPU treatment with respect to our sampling at 51 d (Fig. 3b). Several volatile compounds that were reduced after CPPU treatment in our study are consistent with reductions reported in Shine Muscat by Wang et al.^31^, i.e., α-terpineol, linalool, 2-hexenal (E) at 51 d post treatment and *cis* geraniol at 70 d post treatment. Wang et al.^31^ also reported an increase in hexanol in ‘Shine Muscat’ following CPPU treatment but this was not the case for ‘Sable’ in our study (Table S3). Instead, we observed that CPPU increased other fatty acid derivatives such as decanal, 2-pentenal (E) and 2-hexen-1-ol at 70 d (Fig. 3c). Another important effect of CPPU in our study was the reduction in sesquiterpenes at 51 d (Table S3). However, the significance of this reduction is unclear given the fact that CPPU had no effect on the level of sesquiterpenes at 70 d. The fact that GA increased the levels of rose oxide, α-terpineol, and methyl geranate can strengthen the fruity flavor of the berries^7^.

Given the large number of genes affected by CPPU, it was of interest to further look at a subset of genes that were consistently affected by the treatment at both 51 and 70 d after treatment and in the ‘similar Brix’ group (51 d control and 70 d CPPU). Two main processes were observed: increases in stress-related DEGs with hypoxia as a potential driver and increases in cell wall-related processes potentially associated with cell expansion (Fig. 4d). While there are no physiological studies to support it in our data, hypoxia has previously been associated with ripening and cell death in grape berries^43^ and it can be hypothesized that induction of stress/hypoxia-related genes by CPPU can therefore delay ripening. Mitigation of oxidative stress was attributed to cytokinin by proteins such as glutaredoxin (Fig. 4d)^44^. With respect to cell wall-related genes, induction of *expansins* was described as a hallmark of response to cytokinin^44^ in addition to *laccases* and pectin-modifying genes (Fig. 4d).

There are multiple interactions among plant hormones and changes during ripening. When compared on the basis of similar Brix among the control and the CPPU treatment, it appears that there is repression of several key genes related to hormone signaling (Fig. S2). For example, *AUX/IAA* genes are inhibitors of auxin signaling that is known to delay ripening^45^ while *GID2* inhibits *DELLA* activity and allows response to gibberellins^46^. Other genes with reduced expression upon CPPU treatment were a *BRI1*-associated kinase that controls brassinosteroids signaling and *JAZ* that is a negative regulator of JA signaling^5^. Understanding the complex interaction between external hormone treatment and internal hormone signaling modules requires integration of knowledge from model systems with well-defined genetic resources and physiological data from crop plants.

At the scientific level, this study provides strong evidence for the induction of PA biosynthesis by cytokinin and to its effects on delay of ripening. On the other hand, GA is shown to influence berry size but it does not seem to have a major impact on metabolic and molecular processes including PA biosynthesis. At the practical level, information from this study can be used by growers to better understand how applications of PGRs can modulate berry size, taste, flavor, and color.

## Supplementary information

ANOVA of volatile compounds.

Differentially expressed gene list.

DEGs: Control vs. CPPU 51 d.

DEGs: Control vs. CPPU 70 d.

DEGs: Control 51 d vs. CPPU 70 d.

Supplementary Information
