# Supplementary material for: Cytokinin but not gibberellin application had major impact on the phenylpropanoid pathway in grape
Source: Hortic Res. 2021 Mar 1;8:51. doi: 10.1038/s41438-021-00488-0 (PMC7917099; doi:10.1038/s41438-021-00488-0)
Supplement: Supplementary file 6 — Supplementary Information [file 41438_2021_488_MOESM6_ESM.docx]

**Supplementary Files**


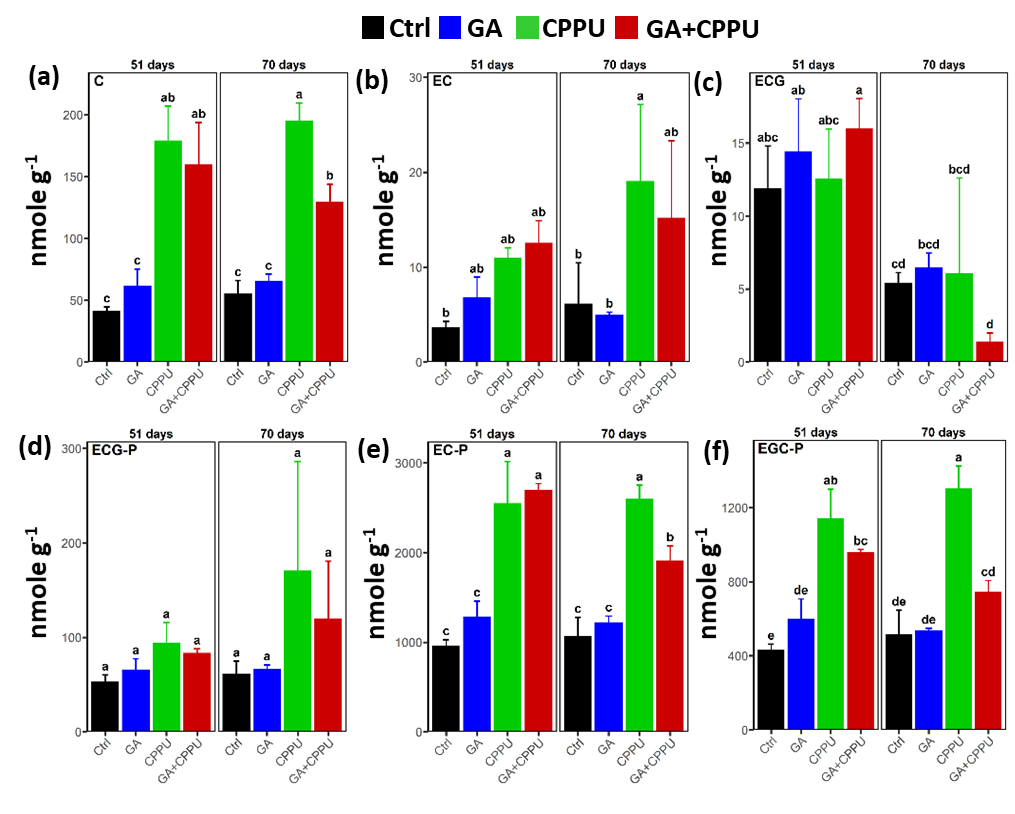


**Fig. S1. Proanthocyanidins (PAs) composition in ‘Sable’ at 51 and 70 d after growth regulator treatments.** The levels of (+)-catechin (C), (−)-epicatechin (EC), epicatechin gallate (ECG), (−)-epicatechin gallate-phloroglucinol (ECG-P), (−)-epicatechin -phloroglucinol (EC-P), (-)-epigallocatechin-phloroglucinol (EGC-P) were determined by RP-HPLC and are expressed as nmole g^-1^ FW **(a-f)**. Values are the mean and standard deviation of 3 replications and different letters above each bar denote significant difference between the treatments by Tukey posthoc test at p≤ 0.05.


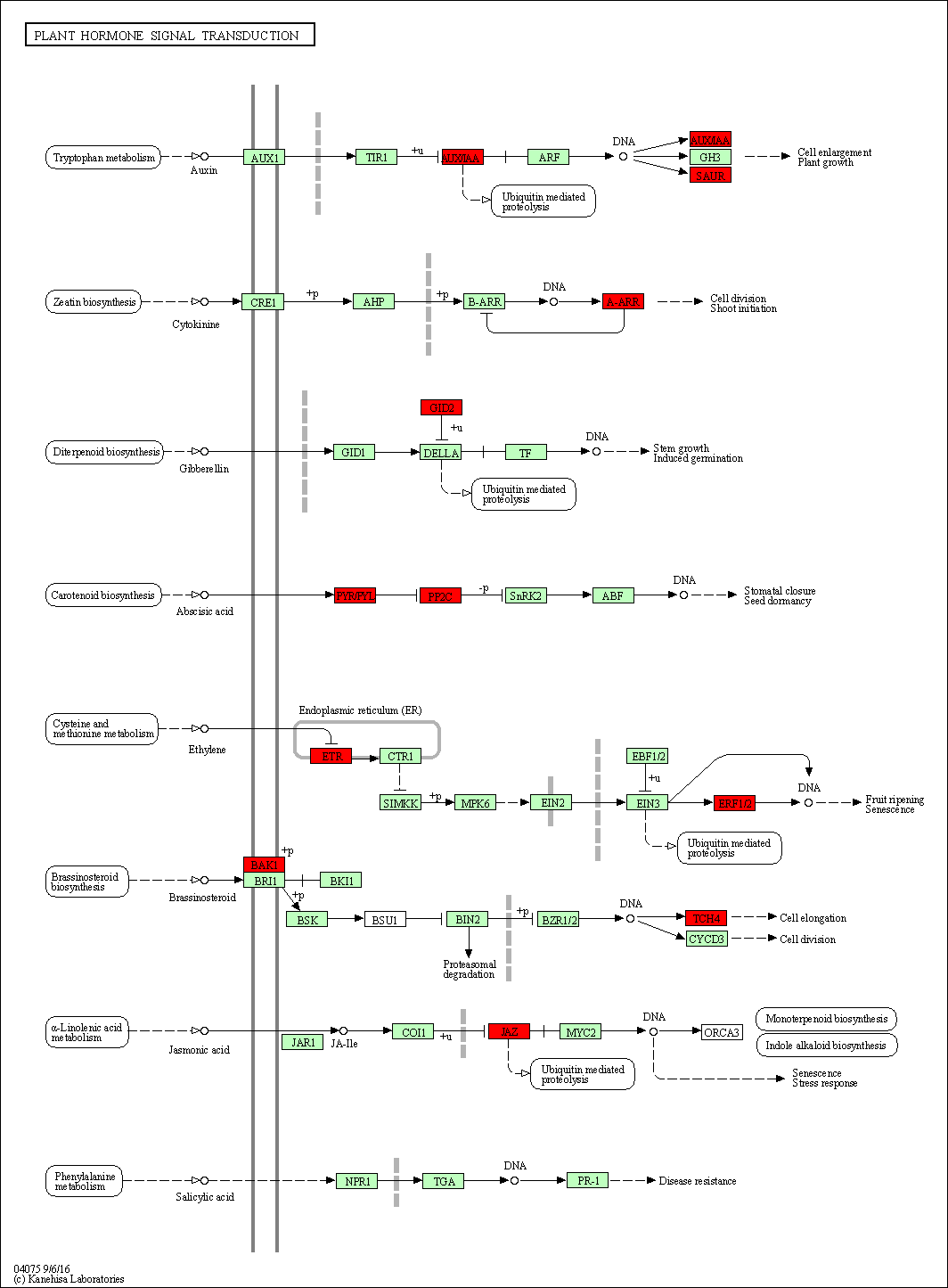


**Fig. S2.** DEGs enrichment of hormone signalling pathway at similar °Brix level using KEGG pathway analysis. Enriched genes in the pathway are with red box.

**Table S1.** Monomeric flavan-3-ols, polymeric phenol, hydroxycinnamate derivatives, flavonols, anthocyanins and polymeric pigment content of ‘Sable’ 51 and 70 d after GA, CPPU and GA+CPPU treatment. Values are mean (±SE) and values with different alphabets differ significantly among treatments according to Student t-test at p ≤ 0.05 and n = 3. All values expressed as mg g−1 FW. Monomeric phenols were analysed using RP-HPLC. ND = not detected. Abbreviations are delph-3-gluc- delphinidin-3-*O*-glucoside; cya-3-gluc - cyanidin-3-*O*-glucoside; pet-3-gluc - petunidin-3-*O*-glucoside; peo-3-gluc - peonidin-3-*O*-glucoside; mal-3-gluc - malvidin-3-*O*-glucoside; delph-3-acetlygluc - delphinidin-3-*O*-acetylglucoside; pet-3-acetlygluc - petunidin-3-*O*-acetylglucoside; peo-3-acetlygluc - peonidin-3-*O*-acetylglucoside; mal-3-acetlygluc - malvidin-3-*O*-acetylglucoside; delph-3-*p*-coumgluc - delphinidin-3-*O*-(6-*p*-coumaroyl) glucoside; pet-3-*p*-coumgluc - petunidin-3-*O*-(6-*p*-coumaroyl) glucoside; peo-3-*p*-coumgluc - peonidin-3-*O*-(6-*p*-coumaroyl) glucoside; mal-3-*p*-coumgluc - malvidin-3-*O*-(6-*p*-coumaroyl) glucoside.

|  | **51 d**ays | | | | **70 d**ays | | | |
| --- | --- | --- | --- | --- | --- | --- | --- | --- |
| **Compound** | Ctrl | GA | CPPU | GA+CPPU | Ctrl | GA | CPPU | GA+CPPU |
| **Monomeric Flavan-3-ols** | | | | | | | | |
| **Epigallocatechin (EGC)** | 0.025±0.003 b | 0.025±0.003 b | 0.025±0.001 b | 0.033±0.005 ab | 0.031±0.002 ab | 0.034±0.005 ab | 0.044±0.002 a | 0.03±0.001 ab |
| **Catechin (C)** | 0.01±0.002 b | 0.014±0.002 b | 0.043±0.004 a | 0.038±0.002 a | 0.009±0.001 b | 0.009±0.001 b | 0.035±0.001 a | 0.031±0.005 a |
| **Epicatechin (EC)** | 0.011±0.003 d | 0.013±0.002 d | 0.031±0.004 c | 0.032±0.004 c | 0.044±0.001 bc | 0.041±0.001 bc | 0.065±0.004 a | 0.051±0.002 b |
| **Polymeric phenol** | 0.376±0.014 c | 0.374±0.003 c | 0.738±0.023 ab | 0.736±0.028 ab | 0.85±0.051 ab | 0.698±0.054 b | 0.892±0.02 a | 0.794±0.037 ab |
| **Hydroxycinnamate derivatives** | | | | | | | | |
| **Caftaric acid** | 0.064±0.002 b | 0.062±0.002 b | 0.083±0.004 a | 0.084±0.002 a | 0.061±0.001 b | 0.057±0.001 | 0.066±0.002 b | 0.059±0.001 b |
| **Caffeic acid** | 0.002±0 a | 0.002±0 a | 0±0 c | 0.001±0 b | 0±0 c | 0±0 c | 0±0 c | 0±0 c |
| **Coutaric acid** | 0.003±0 d | 0.003±0 d | 0.008±0 a | 0.006±0 bc | 0.003±0 d | 0.003±0 d | 0.006±0 b | 0.005±0 c |
| **Ferulic acid** | 0.001±0 bcd | 0.001±0 cd | 0±0 d | 0±0 d | 0.002±0 b | 0.002±0 bc | 0.005±0.001 a | 0.001±0 cd |
| **Monomeric Flavonols** | | | | | | | | |
| **Myricetin glycosides** | 0.162±0.004 abc | 0.167±0.007 ab | 0.102±0.017 d | 0.11±0.001 cd | 0.207±0.012 a | 0.217±0.006 a | 0.174±0.016 ab | 0.15±0.014 bcd |
| **Quer-galactosides** | 0.004±0 a | 0.004±0.001 a | 0.003±0 a | 0.003±0 a | 0.004±0 a | 0.004±0 a | 0.004±0.001 a | 0.003±0 a |
| **Quer-glucosides** | 0.008±0.001 b | 0.005±0 b | 0.012±0.003 ab | 0.005±0 b | 0.009±0 b | 0.006±0 b | 0.018±0.004 a | 0.01±0.002 ab |
| **Quer-rhamnoside** | 0.014±0.001 c | 0.013±0.001 c | 0.006±0.001 d | 0.007±0 d | 0.024±0.001 a | 0.023±0.001 a | 0.019±0.002 ab | 0.016±0.001 bc |
| **Anthocyanins** | | | | | | | | |
| **Del-3-gluc** | 0.413±0.021 a | 0.404±0.022 ab | 0.168±0.009 e | 0.244±0.008 d | 0.338±0.014 bc | 0.314±0.007 c | 0.141±0.006 e | 0.161±0.012 e |
| **Cya-3-gluc** | 0.083±0.003 a | 0.064±0.007 b | 0.02±0.001 c | 0.035±0.002 c | 0.089±0 a | 0.057±0.004 b | 0.02±0.002 c | 0.025±0.002 c |
| **Pet-3-gluc** | 0.402±0.02 a | 0.403±0.019 a | 0.172±0.008 d | 0.253±0.007 c | 0.35±0.014 ab | 0.332±0.007 b | 0.154±0.006 d | 0.174±0.011 d |
| **Peo-3-gluc** | 0.709±0.022 ab | 0.581±0.057 b | 0.184±0.016 d | 0.365±0.025 c | 0.844±0.013 a | 0.6±0.044 b | 0.213±0.023 d | 0.269±0.016 cd |
| **Mal-3-gluc** | 3.044±0.172 ab | 3.497±0.105 a | 1.552±0.065 d | 2.44±0.085 c | 2.97±0.089 b | 3.118±0.043 ab | 1.429±0.048 d | 1.655±0.083 d |
| **Delph-3-acetlygluc** | 0.028±0.002 a | 0.028±0.001 a | 0.012±0.001 d | 0.018±0 c | 0.024±0.001 ab | 0.022±0 bc | 0.012±0 d | 0.013±0.001 d |
| **Pet-3-acetlygluc** | 0.035±0.002 a | 0.036±0.001 a | 0.016±0.001 c | 0.023±0 b | 0±0 d | 0±0 d | 0±0 d | 0±0 d |
| **Peo-3-acetlygluc** | 0.06±0.001 a | 0.044±0.003 b | 0.018±0.001 d | 0.032±0.001 c | 0±0 e | 0±0 e | 0±0 e | 0±0 e |
| **Mal-3-acetlygluc** | 0.406±0.021 b | 0.494±0.005 a | 0.21±0.006 e | 0.339±0.009 c | 0.479±0.013 a | 0.534±0.009 a | 0.274±0.008 d | 0.306±0.012 cd |
| **Delph-3-p-coumgluc** | 0.076±0.004 bc | 0.091±0.001 a | 0.049±0.001 d | 0.067±0.002 c | 0.079±0.002 b | 0.079±0 b | 0.047±0.002 d | 0.047±0.002 d |
| **Pet-3-p-coumgluc** | 0.111±0.005 b | 0.141±0.002 a | 0.08±0.001 c | 0.109±0.003 b | 0±0 d | 0±0 d | 0±0 d | 0±0 d |
| **Peo-3-p-coumgluc** | 0.21±0.003 b | 0.159±0.007 c | 0.06±0.004 d | 0.113±0.006 c | 0.319±0.005 a | 0.233±0.019 b | 0.112±0.015 c | 0.121±0.006 c |
| **Mal-3-p-coumgluc** | 0.895±0.042 c | 1.231±0.017 ab | 0.544±0.013 e | 0.891±0.03 c | 1.187±0.02 b | 1.32±0.009 a | 0.687±0.032 d | 0.758±0.021 d |
| **Polymeric pigment** | 0±0 c | 0±0 c | 0±0 c | 0±0 c | 0.159±0.002 a | 0.159±0.003 a | 0.087±0.002 b | 0.092±0.005 b |

**Table S2**. Characterization of proanthcyanidins from ‘Sable’ at 51 and 70 d after GA, CPPU and GA+CPPU treatment. Values are mean (±SE) and values with different alphabets differ significantly among treatments according to Student t-test at p ≤ 0.05 and n = 3.

|  | **51 d** | | | | **70 d** | | | |
| --- | --- | --- | --- | --- | --- | --- | --- | --- |
|  | **Ctrl** | **GA** | **CPPU** | **GA+CPPU** | **Ctrl** | **GA** | **CPPU** | **GA+CPPU** |
| **Total nmoles** | 1.8±0.1a | 2.5±0.2b | 4.8±0.5c | 4.8±0.1c | 2.1±0.3a | 2.1±0.1a | 4.8±0.2b | 3.3±0.1c |
| **Terminal units** | 57.1±0.8a | 83.1±8.6b | 202.8±17.5c | 188.5±18.6c | 67.1±8.7a | 77.2±3.5a | 220.5±6.7 | 146±12.7 |
| **Extension units** | 1456.9±50.5a | 1953.8±169.4b | 3797.9±369.8c | 3766.1±57.2c | 1650.3±202.3a | 1834.6±46.1a | 4082.4±171.8b | 2796.2±118.6c |
| **Galloyation %** | 4.3±0.2a | 4±0.2a | 2.7±0.1b | 2.5±0b | 3.9±0a | 3.8±0.1a | 4.1±1.4a | 4.1±1.2a |
| **Average MW** | 7922.3±183a | 7346.5±160.2a | 5843.3±63b | 6310.7±538.5ab | 7656.8±96a | 7406.2±192.9a | 5840±231.1b | 6067.5±373.4bc |

Galloylation %= percentage galloylated units (ECG and ECG-P) of the total.

**Table S3.** List of volatile compounds identified in GA, CPPU and GA+CPPU treated berries of “Sable” Seedless using GC-MS. A total of 58 and 67 volatile compounds were identified in berries harvested at 51 and 70 d. In numbers, 31, 31, 33 (at 51 d) and 9, 7, 26 (at 70 d) were significantly differ in treatment compared to the control (Ctrl). Values are mean (±SE) and significantly different values in treatment were highlight in bold with respect to control according to Student t-test at p ≤ 0.05 and n = 3). Volatile compounds were quantified relative to internal standard (2-octanol) and expressed as ng g^-1^ fresh weight of grape berries. Compound identification (ID method) was based on the Retention Index (RI) and Mass Spectra (MS) and authentic standard (Std) when available.

| **Compound** | **Calculated RI** | **Library RI** | **51 d** | | | | **70 d** | | | |
| --- | --- | --- | --- | --- | --- | --- | --- | --- | --- | --- |
|  |  |  | **Ctrl** | **GA** | **CPPU** | **GA+CPPU** | **Ctrl** | **GA** | **CPPU** | **GA+CPPU** |
| Ethanol | #N/A | 448 | 4.79±0.53 | 3.86±0.74 | **0.95±0.14** | **1.49±0.07** | 14.32±0.93 | **20±1.6** | 13.04±1.54 | 14.08±0.16 |
| 2-Butanone | 603.52 | 602 | 2.61±0.21 | 2±0.14 | 2.35±0.1 | **1.4±0.12** | 3.03±0.06 | 3.91±0.46 | 3.41±0.29 | **3.53±0.12** |
| Ethyl acetate | 622.29 | 612 | 0.91±0.09 | 0.9±0.02 | 0.81±0.05 | **0.49±0.11** | 2.23±1.16 | 1.49±0.56 | 4.15±0.29 | 1.55±0.03 |
| Methyl propionate | 640.31 | 621 | 1.44±0.13 | **0.62±0.01** | 1.01±0.16 | **0.76±0.02** | 1.01±0.24 | 0.75±0.07 | 0.8±0.05 | 0.88±0.07 |
| 2-Butanone, 3-methyl- | 667.6 | 666 | 0±0 | 0±0 | 0±0 | 0±0 | 0.36±0.06 | 0.39±0.04 | 0.44±0.04 | 0.4±0.02 |
| 1-Penten-3-ol | 687.35 | 685 | 0.91±0.02 | **0.8±0.02** | 1.23±0.12 | **1.21±0.07** | 1.31±0.11 | 1.25±0.06 | 1.25±0.07 | 1.17±0.09 |
| 1-Penten-3-one | 690.84 | 684 | 6.17±0.04 | **5.25±0.2** | **8.5±0.48** | 7.03±1.16 | 8.97±0.3 | 10.27±0.46 | 9.52±0.97 | 8.37±0.37 |
| Pentanal | 698.74 | 698 | 1.44±0.28 | **3.23±0.48** | **3.61±0.47** | 1.32±0.28 | 2.9±0.3 | 2.13±0.27 | 2.84±0.39 | 2.77±0.2 |
| Butanoic acid, methyl ester | 729.74 | 724 | 1.79±0.12 | 1.58±0.11 | **1.35±0.04** | **1.05±0.11** | 2.94±0.19 | 3.15±0.06 | 3.04±0.34 | 3.31±0.13 |
| 3-Pentanone, 2-methyl- | 760.3 | 726 | 0±0 | 0±0 | 0±0 | 0±0 | 0.27±0.01 | 0.37±0.08 | 0.31±0.05 | **0.38±0.02** |
| 2-Pentenal, (E) | 764.69 | 754 | 2.26±0.08 | 2.11±0.14 | **3.26±0.25** | **3.17±0.3** | 2.82±0.22 | 2.57±0.11 | 3.53±0.33 | 2.6±0.14 |
| (Z)-2-Penten-1-ol | 780.44 | 769 | 0.99±0.03 | 1.39±0.15 | **1.97±0.07** | **1.58±0.06** | 1.76±0.16 | 1.24±0.1 | 1.3±0.1 | 1.5±0.05 |
| Prenol | 785.6 | 778 | 0±0 | 0±0 | 0±0 | 0±0 | 0.57±0.02 | 0.65±0.07 | 0.72±0.1 | **0.85±0.04** |
| Hexanal | 807.84 | 800 | 451.63±14.06 | **347.17±14.9** | **286.02±8.4** | 582.59±81.47 | 710.62±81.42 | 736.09±51.53 | **385.87±27.08** | **422.15±30.23** |
| 2-Hexenal, (E) | 864.83 | 855 | 2322.42±26.97 | 2040.1±107.13 | **2120.79±66.69** | **2994.03±142.54** | 2601.27±196.37 | 1180.94±852.79 | 2313.63±187.57 | 2285.99±157.12 |
| 2-Hexen-1-ol | 873.66 | 862 | 79.65±2.92 | **131.8±6.97** | **57.2±3.15** | **45.63±4.52** | 35.89±2.96 | 42.79±1.48 | **47.93±2.33** | 34.16±1.01 |
| 1-Hexanol | 875.55 | 867 | 69.36±2.31 | **122.5±5.17** | **41.43±2.94** | **25.57±3.33** | 37.21±2.95 | 39.38±1.54 | 31.24±1.11 | 29.86±1.47 |
| 1-Heptanal | 902.83 | 901 | 1.7±0.06 | **1.24±0.06** | 1.56±0.07 | 1.52±0.04 | 2.16±0.11 | 2.13±0.1 | 2±0.06 | 2±0.14 |
| (E,E)-2,4-Hexadienal | 913.84 | 913 | 18.27±0.03 | **12.7±0.67** | **14.8±0.26** | 17.86±0.58 | 28.64±2.47 | 29.08±1.14 | 21.26±1.3 | **20.37±1.58** |
| Methyl hexanoate | 930.19 | 924 | 0.47±0.08 | 0.42±0.05 | 0.39±0.09 | 0.35±0.04 | 1.08±0.14 | 0.79±0.12 | 1.11±0.31 | 0.85±0.04 |
| 2-Heptenal, (Z)- | 961.82 | 958 | 2.2±0.07 | 2.63±0.15 | 4.04±0.49 | 2.31±0.26 | 1.83±0.07 | 1.63±0.26 | **1.21±0.14** | **1.23±0.17** |
| Benzaldehyde | 966.2 | 961 | 1.66±0.33 | 0.9±0.18 | 0.98±0.12 | 1.75±0.38 | 3.09±0.66 | 2.82±0.42 | 2.5±0.3 | 1.63±0.08 |
| 6-Methyl-5-heptene-2-one | 990.65 | 986 | 4.39±0.25 | **7.81±0.6** | 5.73±0.87 | **6.61±0.59** | 7.66±0.72 | 9.33±0.38 | 9.7±0.07 | 8.99±0.42 |
| beta-Pinene | 994.27 | 979 | 3.44±0.15 | 3.64±0.14 | 3.21±0.07 | 3.53±0.43 | 5.96±0.18 | **4.86±0.2** | 5.19±0.51 | **4.76±0.09** |
| 2,4-Heptadienal, (E,E)- | 999.19 | 1007 | 0.37±0.03 | 0.4±0.02 | 0.5±0.07 | **0.8±0.01** | 1.08±0.09 | **0.8±0.04** | **0.66±0.11** | **0.51±0.07** |
| o-Cymene | 1030.66 | 1026 | 0±0 | 0±0 | 0±0 | 0±0 | 0.33±0.05 | 0.2±0.03 | 0.31±0.03 | 0.27±0.01 |
| Limonene | 1035.29 | 1030 | 3.71±0.03 | **3.32±0.02** | **2.99±0.03** | 5.11±0.56 | 16.64±2.29 | 13.77±1.49 | 16.13±5.05 | 12.46±1.03 |
| Benzyl Alcohol | 1041.78 | 1035 | 0±0 | 0±0 | 0±0 | 0±0 | 1.38±0.92 | 0.94±0.16 | 0.39±0.09 | 0.13±0.03 |
| β-trans-Ocimene | 1043.71 | 1041 | 0.43±0.01 | **0.52±0.03** | 0.42±0.05 | 0.43±0.08 | 0.92±0.02 | 0.84±0.04 | 0.86±0.06 | **0.67±0.06** |
| Phenylacetaldehyde | 1049.41 | 1049 | 0±0 | 0±0 | 0±0 | 0±0 | 0.3±0.11 | 0.28±0.08 | 0.18±0.02 | 0.22±0.06 |
| β-cis-Ocimene | 1054.71 | 1051 | 0.43±0.03 | 0.7±0.11 | 0.26±0.13 | 0.49±0.13 | 0.75±0.1 | 0.65±0.13 | 0.64±0.07 | 0.6±0.13 |
| 2-Octenal, (E)- | 1063.56 | 1064 | 2.19±0.16 | 2.28±0.05 | 1.87±0.86 | 1.98±0.2 | 2.22±0.03 | **1.71±0.05** | 1.73±0.16 | 1.72±0.17 |
| 2-Octen-1-ol (E) | 1073.64 | 1073 | 0.77±0.02 | 0.91±0.15 | 1.2±0.62 | **0.63±0.04** | 1.13±0.16 | 0.98±0.1 | 0.77±0.1 | 0.79±0.11 |
| 1-Octanol | 1075.97 | 1075 | 1.43±0.13 | 1.09±0.11 | **0.75±0.06** | 1.21±0.2 | 1.16±0.31 | 0.83±0.02 | 1.03±0.16 | 0.64±0.08 |
| Linalool Oxide (isomer I) | 1078.57 | 1077 | 1.24±0.1 | 1.31±0.21 | **0.76±0.03** | **0.97±0.07** | 1.46±0.36 | **2.63±0.27** | 1.93±0.45 | 2.32±0.06 |
| cis-Linalool Oxide | 1092.64 | 1091 | 0.49±0.03 | 0.6±0.08 | 0.44±0.13 | 0.38±0.02 | 0.77±0.11 | 1.16±0.08 | 1.02±0.14 | 0.96±0.02 |
| Linalool | 1102.17 | 1100 | 3.54±0.11 | 3.48±0.21 | 2.87±0.22 | 4.24±0.39 | 18.87±5.48 | 35±5.03 | 28.6±10.19 | 22.81±2.21 |
| Nonanal | 1107.54 | 1104 | 8.53±0.36 | 7.51±0.09 | **7.03±0.1** | **6.46±0.2** | 5.02±0.41 | 5.79±0.51 | 6.39±0.36 | **7.69±0.12** |
| trans-Rose oxide | 1114.6 | 1115 | 1.01±0.09 | 1.09±0.09 | **0.67±0.07** | 0.85±0.03 | 1.04±0.06 | **1.61±0.09** | 0.82±0.08 | **1.27±0.02** |
| Octanoic acid, methyl ester | 1129.41 | 1126 | 0.22±0.03 | **0.04±0.02** | **0.05±0.02** | **0.06±0.03** | 1.02±0.19 | 1.22±0.11 | 1.16±0.42 | 0.73±0.03 |
| cis-Rose oxide | 1133.79 | 1134 | 0±0 | 0±0 | 0±0 | 0±0 | 0.36±0.02 | 0.49±0.06 | 0.23±0.07 | 0.37±0.03 |
| 2,6-Nonadienal, (E,Z)- | 1159.52 | 1156 | 0.44±0.03 | 0.53±0.03 | 0.32±0.03 | 0.3±0.06 | 0.66±0.01 | 0.58±0.06 | 0.44±0.12 | **0.3±0.04** |
| 2-Nonenal, (E)- | 1165.17 | 1162 | 1.46±0.14 | **1.92±0.06** | 1.27±0.07 | **0.82±0.13** | 1.46±0.08 | 1.19±0.11 | 1.52±0.1 | 1.2±0.14 |
| Epoxylinalol 1 | 1175.36 | 1173 | 0.88±0.06 | 1.07±0.05 | 1.01±0.1 | **1.34±0.13** | 1.46±0.44 | 1.67±0.05 | 1.67±0.31 | 1.16±0.06 |
| Epoxylinalol 2 | 1179.01 | 1179 | 0.3±0.03 | 0.34±0.06 | 0.3±0.04 | 0.23±0.02 | 0.74±0.21 | 1.27±0.04 | 0.86±0.24 | 0.6±0.01 |
| α-Terpineol | 1195.36 | 1195 | 0.65±0.01 | 0.57±0.07 | **0.28±0.07** | 0.85±0.06 | 0.73±0.1 | **1.18±0.09** | 1.17±0.26 | **2.42±0.09** |
| Decanal | 1208.97 | 1208 | 1.99±0.1 | **1.58±0.09** | 1.9±0.02 | **1.24±0.17** | 1.56±0.14 | 1.23±0.07 | 1.67±0.1 | 1.15±0.12 |
| β-Cyclocitral | 1227.93 | 1226 | 0.42±0.04 | 0.46±0.05 | 0.32±0.02 | 0.68±0.12 | 0.59±0.07 | 0.61±0.02 | 0.61±0.1 | 0.5±0.05 |
| Nerol | 1234 | 1236 | 9.66±0.24 | **16.31±0.51** | **7.79±0.09** | 10.14±2.15 | 26.34±2.14 | **16.76±1.55** | **12.83±0.53** | **12.23±0.63** |
| Isogeraniol | 1239.3 | 1273 | 0±0 | 0±0 | 0±0 | 0±0 | 2.11±0.34 | 1.33±0.09 | **0.7±0.15** | **0.82±0.11** |
| β-Citral | 1247.33 | 1244 | 0.89±0.04 | **1.64±0.01** | 0.7±0.11 | 0.87±0.09 | 1.72±0.29 | 0.99±0.09 | 0.88±0.03 | **0.74±0.07** |
| cis-Geraniol | 1261.04 | 1258 | 35.47±1.85 | **60.79±1.66** | 39.11±2.38 | 52.73±20.54 | 48.17±6.86 | 20.25±0.11 | 33.74±4.71 | **23.99±2.91** |
| Geranial | 1277.07 | 1273 | 1.91±0.04 | **3.5±0.07** | 1.85±0.35 | 2.32±0.51 | 3.06±0.51 | 1.64±0.28 | 1.7±0.28 | **1.35±0.11** |
| Methyl geranate | 1331.16 | 1321 | 0.57±0.12 | **1.09±0.03** | 0.6±0.06 | **1.09±0.07** | 8.44±0.19 | **10.76±0.34** | 9.61±0.46 | **10.27±0.48** |
| δ-Elemene | 1348.57 | 1339 | 1.54±0.06 | **0.87±0.19** | **0.11±0.05** | **0.2±0.09** | 0.25±0.02 | 0.3±0.04 | 0.44±0.08 | 0.31±0.03 |
| α-Cubebene | 1360.99 | 1354 | 1.46±0.01 | **0.92±0.08** | **0.23±0.07** | **0.43±0.04** | 0.33±0.02 | 0.44±0.08 | 0.46±0.07 | **0.43±0.02** |
| Ylangene | 1383.5 | 1374 | 10.49±0.39 | **4.94±0.93** | **0.63±0.25** | **1.55±0.38** | 2.92±0.16 | 3.72±0.5 | 3.06±0.66 | 3.15±0.12 |
| β-Bourbonene | 1396.33 | 1391 | 8.87±0.38 | **4.28±0.9** | **0.21±0.11** | **0.63±0.17** | 1.94±0.22 | 3.07±0.42 | 2.13±0.39 | **3.07±0.27** |
| β-Caryophyllene | 1433.94 | 1428 | 8.88±0.14 | **3.71±0.77** | **0.58±0.33** | **1.15±0.3** | 1.66±0.13 | 2±0.26 | 2.75±0.56 | **2.14±0.05** |
| α-Guaiene | 1450.03 | 1439 | 0±0 | 0±0 | 0±0 | 0±0 | 0.13±0.01 | 0.14±0.05 | 0.22±0.04 | 0.14±0.02 |
| α-Caryophyllene | 1467.63 | 1460 | 0.8±0.03 | **0.44±0.04** | **0.1±0.03** | **0.22±0.03** | 0.34±0.03 | 0.29±0.04 | 0.37±0.05 | **0.26±0** |
| γ-Muurolene | 1486.99 | 1485 | 1.44±0.09 | 0.77±0.22 | **0.08±0.02** | **0.09±0.02** | 0.21±0.05 | 0.28±0.02 | 0.36±0.09 | 0.34±0.05 |
| β-Guaiene | 1496.85 | 1491 | 1.01±0.04 | **0.51±0.09** | **0.1±0.04** | **0.29±0.13** | 0.18±0.06 | 0.2±0.06 | 0.28±0.02 | 0.23±0.02 |
| β-Cadinene | 1508 | 1519 | 9.9±0.38 | **5.16±0.95** | **0.55±0.35** | **1.48±0.47** | 1.64±0.05 | 2.25±0.27 | 2.94±0.61 | **2.34±0.12** |
| γ-Cadinene | 1524.5 | 1524 | 9.39±0.49 | **4.63±1.02** | **0.31±0.28** | **0.95±0.32** | 1.83±0.11 | 2.46±0.32 | 2.39±0.44 | 2.32±0.15 |
| δ-Cadinene | 1532.64 | 1528 | 8.04±0.23 | **4.39±0.97** | **0.38±0.29** | **1.23±0.41** | 1.44±0.04 | 1.88±0.22 | 2.28±0.41 | **2±0.13** |
| Manoyl oxide | 1983 | 1983 | 3.75±0.26 | **1.53±0.28** | **0.09±0.03** | **0.18±0.05** | 4.76±0.64 | 3.57±0.54 | **1.97±0.17** | **1.88±0.08** |

**Supplementary Excel data files**

**Table S4.** Analysis of variance (ANOVA) showing that 62 of the 68 volatile compounds present at 51 and 70 d differed in content by treatments or time. Volatile compounds were analyzed by GC-MS. FDR and P values ≤ 0.05.

**Table S5.** List of DEGs among GA, CPPU and control at 51 and 70 d. A total of 3801 genes were differentially expressed among the treatments.

**Table S6.** List of upregulated and downregulated genes among control and CPPU at 51 d.

**Table S7.** List of upregulated and downregulated genes among control and CPPU at 70 d.

**Table S8.** List of upregulated and downregulated genes among control at 51 d and CPPU at 70 d at similar sugar level.
